# Excavators

**Published:** 1857-03

**Authors:** J. Taft


### EXCAVATORS.

BY J. TAFT.

By this name we designate not only all those small cutting instruments used for removing the decay from the cavity, and giving to it its proper form, but also those employed in cutting away portions of the teeth in separation; and for opening up the orifices of cavities.

The first we shall describe, is the heavy cutting instrument. These are of the thick chisel shape. They should be made of good steel, well worked and perfectly tempered. Every step in the manufacture should be most perfectly executed to insure an edge that will cut, not only dentine, but enamel, the hardest animal substance. Various sizes of the straight chisel form are required. In all cases they should be as thick as possible, without interfering with their efficiency; so firm that there will be no springing or tremulous motion under the pressure they are required to sustain. For the separation of the front teeth, they should be so thin as to pass readily into the required space, and about one-fourth of an inch in width upon the edge. For the separation of the bicuspids and molars, the instrument should be much thicker and broader. They should be as thick as the respective separation will admit. It in some instances should have the edge oblique, in others square.

It is seldom that any curve is required upon these instruments, and it is always better to use a straight instrument, if the point upon which it is to act does not require a curved one. Occasionally a little anterior curve will be required in the shaft of the instrument to facilitate the approach of the instrument in a small mouth, to the anterior approximal surface of a second or third molar.

A heavy instrument with a sharp point and lateral curvature can be often used efficiently for opening up cavities and cutting down strong projections of enamel. This class of instruments we consider as valuable as any other instruments on our case; and every operator should have at hand sufficient variety to meet every case.

BUR DRILLS.—This is an indispensible class of instruments. There are various forms of these. They should be made of the best steel, and wrought very carefully. After forging as near the proper size and form as possible, the bulb is made by dressing with a fine file; this, however, does not secure the most perfect form, as it is very difficult to make it perfectly true with the file. To secure the most perfect instrument, the bulb should be turned in a lathe; those made in that manner cut much more smoothly; they do not catch and jar as those of less regular form.

After the bulb is formed, it is cut, usually with a sharp edged file.

These instruments partake of various forms. In one, the bulb is a perfect sphere; and again, there is the cone shape, with various degrees of bevel, terminating in a sharp point. Some have a perfectly cylindrical form, cut upon their sides and end; others are of the form of a wheel, some cut only on the edge or circumference, others cut both on the edge and end. The cut upon all of these should be very regular and uniform. The cutting is usually done by hand, but should be done by machinery. There should be a variety in size of all these, from considerably less than the smallest cavity the dentist ever attempts to fill—the smallest about one thirty-second of an inch in diameter; the largest about one-fifth of an inch. Including these extremes, there should be about fifteen sizes of each particular form.

These are used for opening cavities. With them a more regular and perfect orifice is made in small and medium cavities, when they can be approached, than by any other method.

They are also used to some extent for forming cavities, and even sometimes in large cavities for making retaining points for a filling.

COMMON DRILLS.—These drills are of various forms; some are square upon the points, others are spear-shaped, the point of various angles. To make the edge, the point is dressed from one or both sides. When dressed wholly from one side, the drill only cuts when being turned one way. These instruments may be used for forming the bottom of some cavities. They are used by some operators for forming retaining points in cavities to be filled.

The burs and drills may be made of pieces of wire one and a half inches long, and fitted to a socket handle that will accommodate a large number; or they may be made of a continuous piece of large wire. The latter is the preferable method, as much time is consumed in changing them in sockets.

The handles should be made six or eight sided, and cut on each alternate side.

In using these instruments the socket ring is almost indispensable. This is an open ring, to fit the middle or index finger, with a socket attached in which the end of the instrument rests while in use.

The drill is rotated with the fingers and thumb.

Various other methods have been employed for rotating drills. The bow, and numerous spiral fixtures and appliances have been used, almost all of which, so far as practical results are concerned, are useless.

BROACHES.—The three or four sided broaches are made of various sizes, of the best steel. They are made both straight and tapered. They should in no case have more than a spring temper, in which the color, when drawing, is a deep blue.

These instruments are used for enlarging the canals of fangs for filling. Six or eight sizes will probably meet all ordinary cases. When the course into the canal is tortuous the temper of the instrument should be so low that it will bend.

THE SMALL CUTTING INSTRUMENTS.—Of the small cutting instruments for opening up, removing decay from and forming cavities, there is a great variety, though a very few general principles would embrace the whole.

There has hitherto been no very systematic arrangement of these instruments. Such, however, would be very convenient, both for the profession and for the manufacturers of dental instruments. The following classification we have adopted, and found it very convenient:

The classes are arranged by numbers, placing in the first class the least complicated and most simple forms, and with each number taking a more complicated form.

All the varieties are embraced in ten numbers. These take into consideration the form of the points and their position upon the shaft to which they are attached, and not any curvature of the shaft, at any distance from the point.

No. 1 is simply a flat point slightly curved, with a round edge transverse to its shaft. Four sizes will be sufficient for ordinary purposes.

No. 2 is a flat point with a short curve bringing the point to a right angle with the shaft; the edge is transverse. This differs from No. 1 in its short abrupt curve, and the edge is more nearly square. Four sizes.

No. 3 is a flat point with a square, transverse edge, and arises at a right angle from the shaft; the blade from one to two lines in length. Four sizes.

No. 4 is a flat point curved till the point is at a right angle with the shaft; the edge is parallel with the shaft; the blade from the center of the curve to the edge, from one and a half to three lines; the edge straight. Four sizes.

In each of the above the edges should expand slightly in width.

No. 5 is a flat point with a square edge, which is parallel with the shaft, and arises at a right angle from it. The blade from one-half to two and a half lines in length, and from one-half to one line in width, and no expansion at the edge. Six sizes.

Nos. 6 and 7 are right and left excavators, with flat points and double curves. First curve to an angle of about twenty degrees; then the points curved laterally, right and left, with a regular curve from the first curve to the point. Length of the blade from two to five lines. Four sizes.

No. 8 is a crescent shaped point, the blade arises by a small attachment from the shaft, and is a right angle with it. The edge is a regular curve, describes about two-fifths of a circle, and is parallel with the handle. The points should be perfectly formed. Six sizes.

No. 9. The form of the point is the same as No. 8; the difference is in the position of the blade; its edge is transverse to the shaft, and rises from it at an angle of one hundred and thirty degrees. Six sizes.

No. 10. The point has the same shape as Nos. 8 and 9.

The cutting edge is transverse to the shaft, and rises by a small neck at a right angle from it. Six sizes.

These embrace the most important forms for excavators. Modifications will be required for particular cases. Some of these Nos., 8, 9 and 10, are not extensively used. A few have been using them for some years, and prize them so highly that they would not consent to be without them. In the formation of many difficult points they are far more applicable than any other instrument we have. For instance, in the formation of the cervical wall of an approximal cavity of any of the teeth, but particularly of the superior bicuspids and molars, there is no instrument so applicable and efficient as No. 9; with it that part of the cavity, so frequently neglected, is just as easily formed as any other.

Cases will be presented occasionally, in which some curvature of the shaft of the instrument will be required. No more curve should be given than is necessary to meet the case. It is impossible to manipulate with the same precision and delicacy with curved as with straight instruments. These curves will be modified by the position of the decay upon the tooth and the location in the mouth.

